# Risk assessment of toxic residues among some freshwater and marine water fish species

**DOI:** 10.3389/fvets.2023.1185395

**Published:** 2023-07-25

**Authors:** Mohamed A. Hussein, Nanis S. Morsy, Abdallah F. Mahmoud, Wageh S. Darwish, Mohamed T. Elabbasy, František Zigo, Zuzana Farkašová, Ibrahim F. Rehan

**Affiliations:** ^1^Food Control Department, Faculty of Veterinary Medicine, Zagazig University, Zagazig, Egypt; ^2^College of Public Health and Molecular Diagnostics and Personalized Therapeutics Center (CMDPT), Hail University, Hail, Saudi Arabia; ^3^Department of Nutrition and Animal Husbandry, University of Veterinary Medicine and Pharmacy, Košice, Slovakia; ^4^Department of Husbandry and Development of Animal Wealth, Faculty of Veterinary Medicine, Menoufia University, Shebin Alkom, Egypt; ^5^Department of Pathobiochemistry, Faculty of Pharmacy, Meijo University Yagotoyama, Nagoya, Japan

**Keywords:** lead, cadmium, mercury, arsenic, fish, daily intakes, risk assessment index, Egypt

## Abstract

Egypt has several beaches, as well as the Nile River and a few lakes; therefore, it could compensate for the lack of protein in red meat with fish. Fish, however, may become a source of heavy metal exposure in humans. The current study was to assess the level of five toxic metals, lead (Pb), cadmium (Cd), mercury (Hg), arsenic (As), and aluminum (Al), in six species, namely, *Oreochromis niloticus* (*O. niloticus*)*, Mugil cephalus* (*M. cephalus*)*, Lates niloticus* (*L. niloticus*), *Plectropomus leopardus* (*P. leopardus*), *Epinephelus tauvina* (*E. tauvina*)*, and Lethrinus nebulosus* (*L. nebulosus*), collected from the El-Obour fish market in Egypt. The residual concentrations of the tested toxic metals in the examined *O. niloticus, M. cephalus, L. niloticus, E. tauvina, P. leopardus*, and *L. nebulosus* species were found to be higher than the European Commission's maximum permissible limits (MPL) for Pb and Cd by 10 and 20%, 15 and 65%, 75 and 15%, 20 and 65%, 15 and 40%, and 25 and 5%. In contrast, 30% of *L. niloticus* exceeded the MPL for Hg. It was shown that the average estimated daily intake (EDI) and the target hazard quotient (THQ) in fish samples are below safety levels for human consumption and hazard index (HI < 1). From the human health point of view, this study showed that there was no possible health risk to people due to the intake of any studied species under the current consumption rate in the country.

## Introduction

Fish gives customers animal protein with a high biological impact while simultaneously overcoming the challenge of red meat scarcity. Fish is one of the most significant protein sources in Egypt (1). In addition, seafood is endorsed as part of a fit diet in the majority of dietary guidelines (2). The potential toxicity of pollutants, including mercury, lead, cadmium, and arsenic, which may be present in fish and seafood products, has recently caused public health concerns. Heavy metals cannot be biodegraded; instead, bioaccumulation can increase their concentration (3, 4). Both natural and manmade sources release these metals into the environment (5). Heavy metals may therefore accumulate in the food chain and endanger the health of consumers (6). It is suggested that variations in the absorption and depuration times of specific metals are the primary cause of metal bioaccumulation in fish. Many factors, including the time of year, the physical and chemical properties of the water, industrial development, fertilizers, livestock manure, air pollution, mining, and excessive pesticide use, can lead to metal accumulation in different fish tissues (7). There are a number of harmful effects that heavy metals have on human health. Lead is considered one of the toxic metals, for instance, that has been related to multiple instances of child mortality, including those in China and Zambia (8, 9). Additionally, the effects of Pb on mental health and intelligence are harmful. Heavy metal cadmium (Cd) has no known physiological purpose. Itai-Itai disease, which has been linked to heavy fish consumption in Japan, is primarily caused by cadmium. Such a condition is characterized by osteomalacia and kidney dysfunction (10). Additionally, Cd is regarded as a group B1 carcinogen (11). Another heavy metal, arsenic, has also been connected to numerous organ damage, unidentified processes of carcinogenesis, and skin irritation (12). The third most common element in the crust of the Earth is aluminum (Al), which is widely distributed throughout the ecosystem and, in particular, in the food chain and is regarded as a non-essential metal that causes urological problems as well (13). For the sake of human health, it is crucial to assess the chemical quality of aquatic species, particularly the number of heavy metals in fish (14). Because Egypt has large beaches, as well as weak poultry and red meat production, owing to its reliance on yellow grain supplied by Ukraine, consuming fish is one of the alternatives available to customers. Consequently, the present study aimed to detect the concentrations of some metals (As, Cd, Pb, Hg, and Al) in the examined *O. niloticus, M. cephalus, L. niloticus, E. tauvina, P. leopardus*, and *L. nebulosus* species. In addition, a non-carcinogenic health risk assessment was carried out on the Egyptian population.

## Materials and methods

### Sample collection

**Figure 1:**
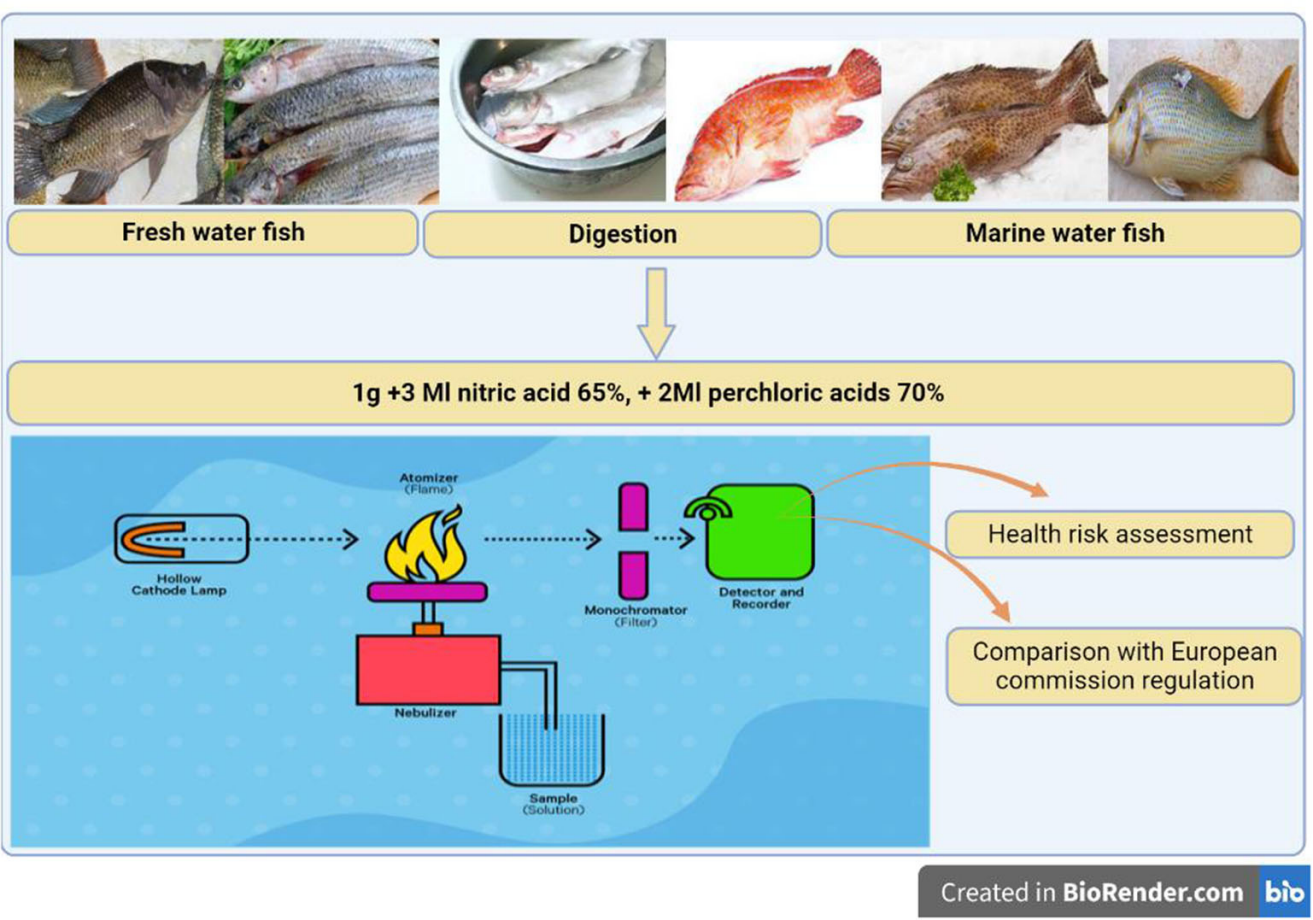
The schematic cartoon of the experimental strategy—designed by BioRender.com.

In total, 120 freshwater and marine water fish were obtained from El-Obour city fish market, Egypt. Fish samples were chosen from apparently freshwater fish that showed no signs of deterioration. From February to August 2022, six fish species (20 of each) were collected including: Three fresh water fish (*O. niloticus, M. cephalus, L. niloticus*) and three marine water fish (*E. tauvina, P. leopardus, L. nebulosus*). The schematic cartoon of the experimental design is represented in Figure 1.

### Sample preparation

Determination of different metals was made as recommended by the previous report (15). Fish samples were chosen from apparently freshwater fish that showed no signs of deterioration. Fish samples were thawed, headed, and eviscerated with stainless steel scalpels, the flesh (edible part) was ground and then homogenized in a domestic food blender, 1 gram of the sample mixture was placed in a 5-mL digestion solution composed of 3:2 parts of nitric acid (65%) and perchloric acid (70%), respectively, for 12 h, and the homogenate was left at room temperature. In a water bath, the mixture was then heated for 3 h at 70°C with stirring every 30 min. The digested mixture was diluted with 20 mL DDW, brought to room temperature, and then filtered through a filter paper. Up until the measurement of heavy metals, the filtrate was left at 20°C.

### Analytical procedures

Pb, Cd, and Al were measured using a graphite furnace atomic absorption spectrophotometer (PerkinElmer^®^ PinAAcleTM 900T Atomic Absorption Spectrophotometer; Shelton, CT, USA).

### Quality assurance and quality control

For ensuring the accuracy of the analytical processes, the National Research Council of Canada's DORM-3 Fish Protein reference material was used. The detection limits (μg/g) for Pb, Cd, As, Hg, and Al were 0.01, 0.005, 0.02, 0.01, and 0.10, respectively. The measured amounts for the metals under test were expressed in microgram/gram wet weight.

### Estimated daily intake

According to the U.S. Environmental Protection Agency (US EPA) (16), the EDI values of the heavy metals that the Egyptian population consumed through the consumption of canned meat and chicken products were determined using the following equation:

EDI (μg/kg/day) = C_m_ X F_IR_/BW, where FIR is the rate at which Egyptians consume fish, and Cm is the concentration of the analyzed metal (g/g wet weight). FIR was set at 48.57 g of flesh per day (17), while BW was set at 70 kg for Egyptian adults.

### Health risk assessment

The hazard quotient (HQ) of the evaluated heavy metals was determined according to US EPA (16) using the following formula:

HQ = EDI/RFD X 10^−3^. Meanwhile, for lead, we used risk index (RI) = EDI x SF.

RfD is the oral reference dose; the RfD values for Pb, Cd, As, Hg, and Al were 0.004, 0.001, 0.0003, 0.0005, and 1 mg/kg/day, respectively (18, 19). The oral slope factor (SF) for lead was 0.0085 mg/kg/day (20).

The risk of combined metals was calculated using a hazard index (HI). HI was derived from the equation as follows: HI = THQ (Pb) + THQ (Cd) + THQ (As) + THQ (Hg) + THQ (Al). When HQ or HI value is >1, it means human exposure to risk (16). In addition, the RI was considered insignificant if the RI was < 10^−6^; the RI was considered allowable or tolerable if RI was 10^−6^ < RI < 10^−4^; and the RI was considered significant if the RI was >10^−4^ (21).

### Statistical analysis

ANOVA, the *post-hoc* test, and the Turkey–Kramer HSD difference test (JMP) were used to evaluate the statistical analysis (SAS Institute, Cary, NC, USA). A *P*-value of 0.05 was regarded as significant. The means and standard deviation were used to express the values (SD) (22).

## Results

### Lead residues

**Figure 2:**
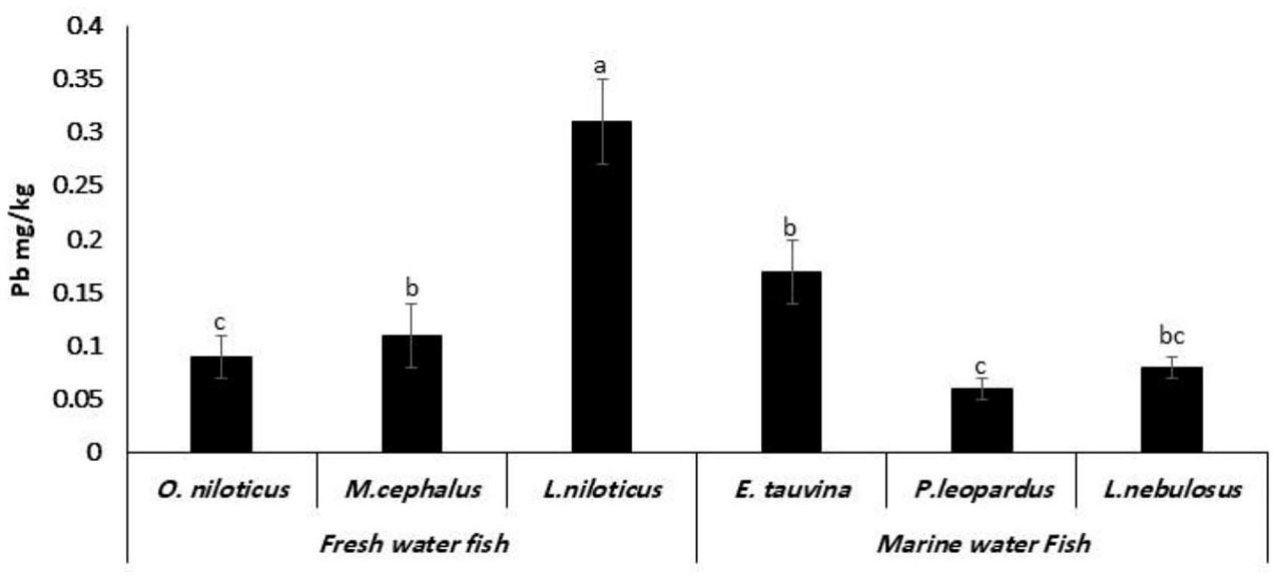
Lead (Pb) residues (mg/kg wet weight) in fresh water and marine water fish. ^a, b, c^Columns bearing different small letters were statistically significant at a *P*-value of < 0.05.

Figure 2 shows the achieved results that Pb was detected in all examined fish species. *L. niloticus* had the significantly highest Pb content of 0.31 ± 0.04 mg/kg, followed by *E. tauvina* (0.17 ± 0.03 mg/kg), *M. cephalus* (0.11 ± 0.03 mg/kg), *O. niloticus* (0.09 ± 0.02 mg/kg), *L. nebulosus* (0.08 ± 0.01 mg/kg), and *P. leopardus* (0.06 ± 0.01 mg/kg).

### Cadmium residues

**Figure 3:**
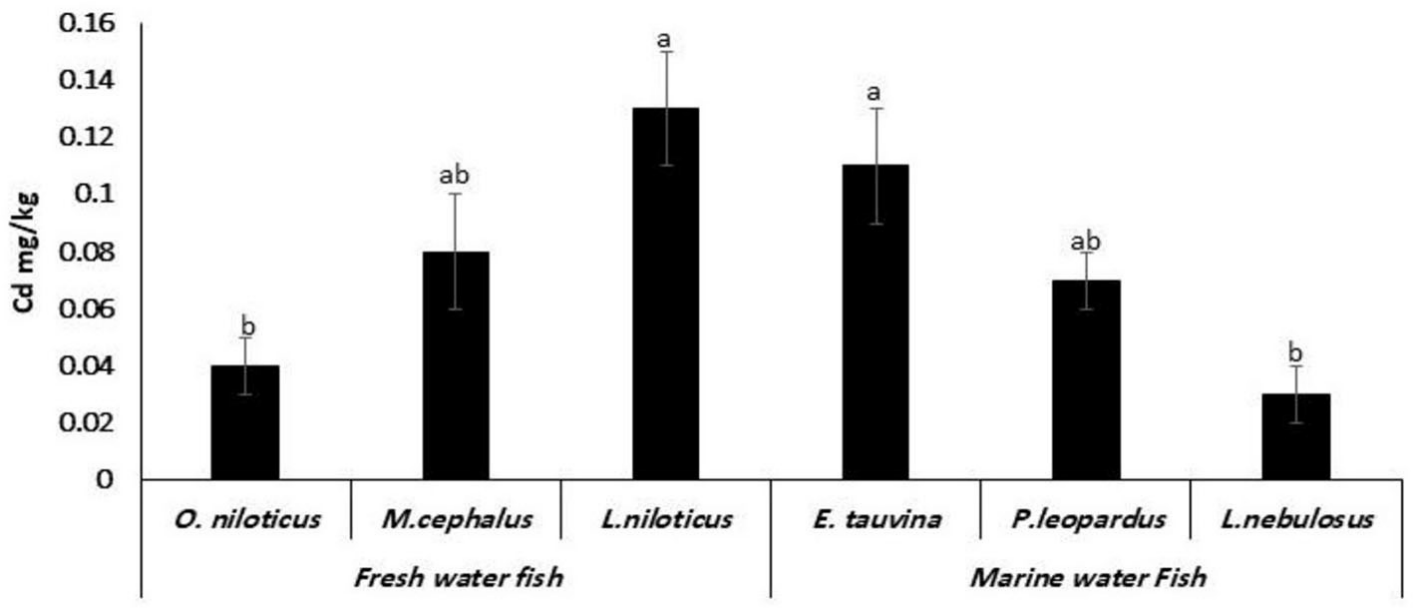
Cadmium (Cd) residues (mg/kg wet weight) in fresh water and marine water fish. ^a, b, c^Columns bearing different small letters are statistically significant at a *P*-value of < 0.05.

Cadmium was found in all species of fish that were studied. *L. niloticus* had significantly (*P* < 0.05) higher Cd content of 0.13 ± 0.02 mg/kg, followed by *E. tauvina, M. cephalus, P. leopardus, O. niloticus*, and *L. nebulosus* with the mean Cd content of 0.11 ± 0.02, 0.08 ± 0.02, 0.07 ± 0.01, 0.04 ± 0.01, and 0.03 ± 0.01 mg/kg, respectively (Figure 3).

### Arsenic residues

**Figure 4:**
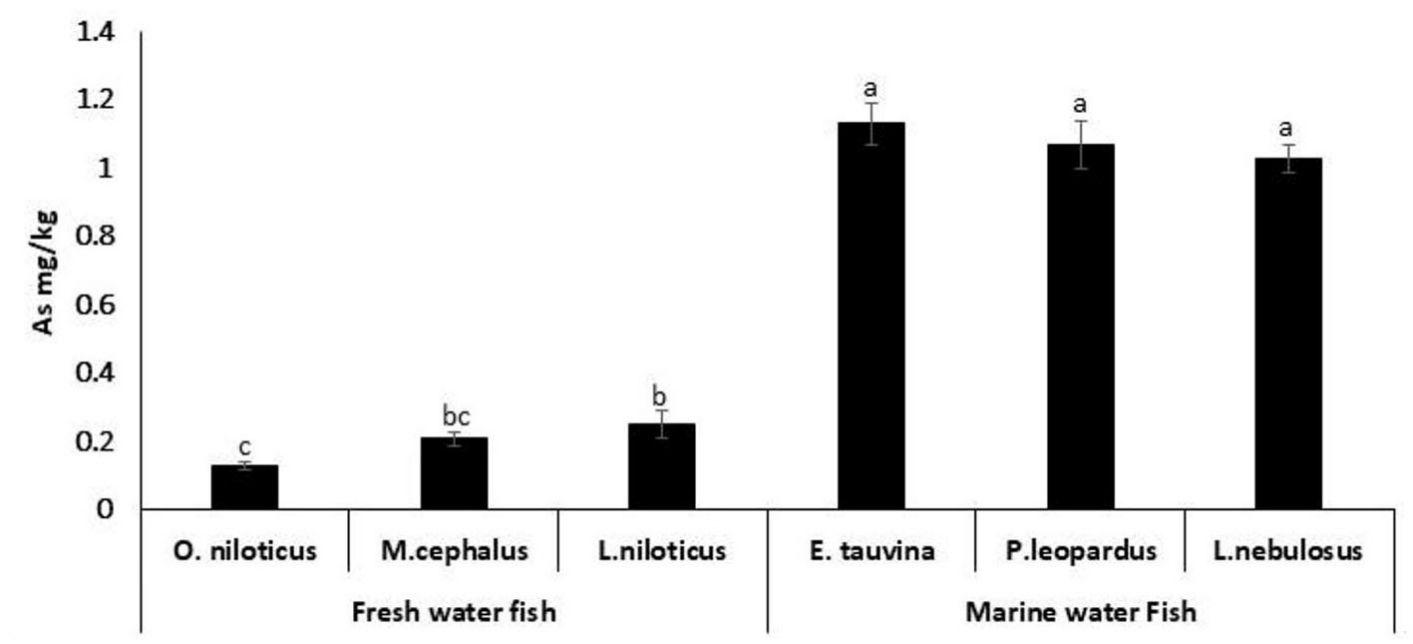
Arsenic (As) residues (mg/kg wet weight) in fresh water and marine water fish. ^a, b, c^Columns bearing different small letters are statistically significant at a *P*-value of < 0.05.

Arsenic residues were recorded in all fish species with the mean values of 0.13 ± 0.01, 0.21 ± 0.02, 0.25 ± 0.04, 1.13 ± 0.06, 1.09 ± 0.07, and 1.03 ± 0.04 mg/kg in the examined *O. niloticus, M. cephalus, L. niloticus, E. tauvina, P. leopardus, and L. nebulosus* species, respectively (Figure 4).

### Mercury residues

**Figure 5:**
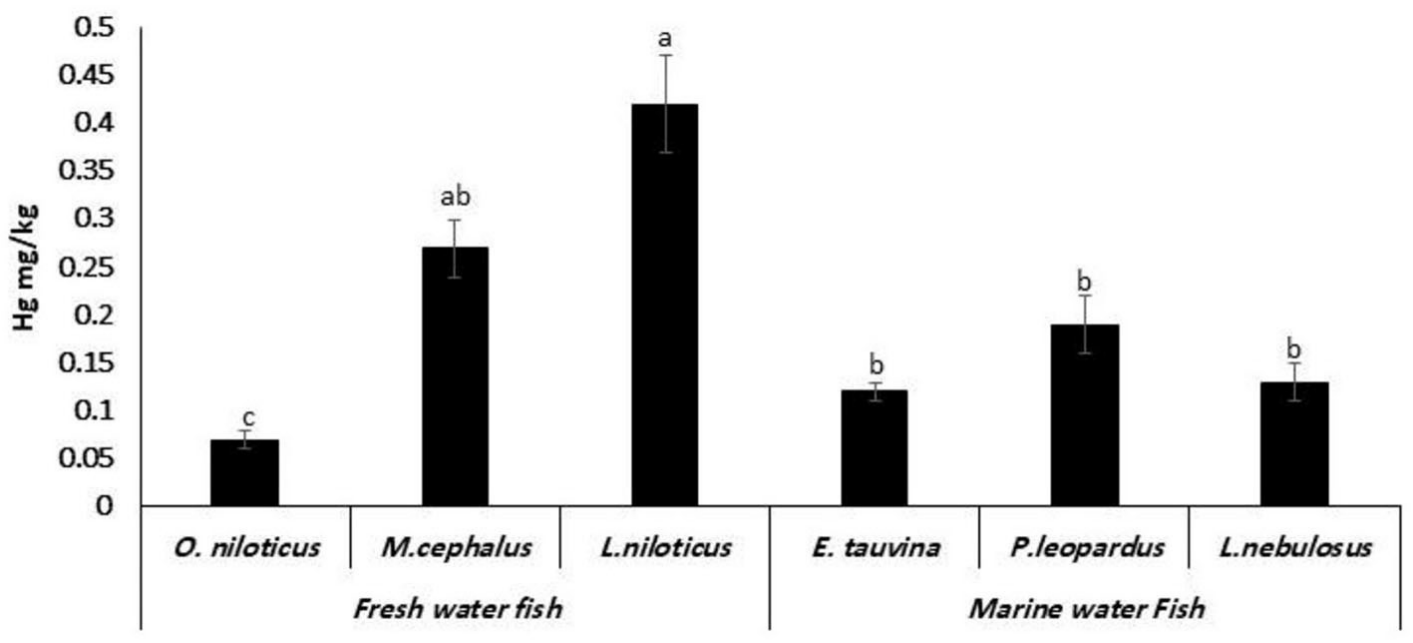
Mercury (Hg) residues (mg/kg wet weight) in fresh water and marine water fish. ^a, b, c^Columns bearing different small letters are statistically significant at a *P*-value of < 0.05.

The mean values of Hg were 0.07 ± 0.01, 0.27 ± 0.03, 0.42 ± 0.05, 0.12 ± 0.01, 0.19 ± 0.03, and 0.13 ± 0.02 mg/kg in the examined *O. niloticus, M. cephalus, L. niloticus, E. tauvina, P. leopardus*, and *L. nebulosus* species, respectively (Figure 5).

### Aluminum residues

**Figure 6:**
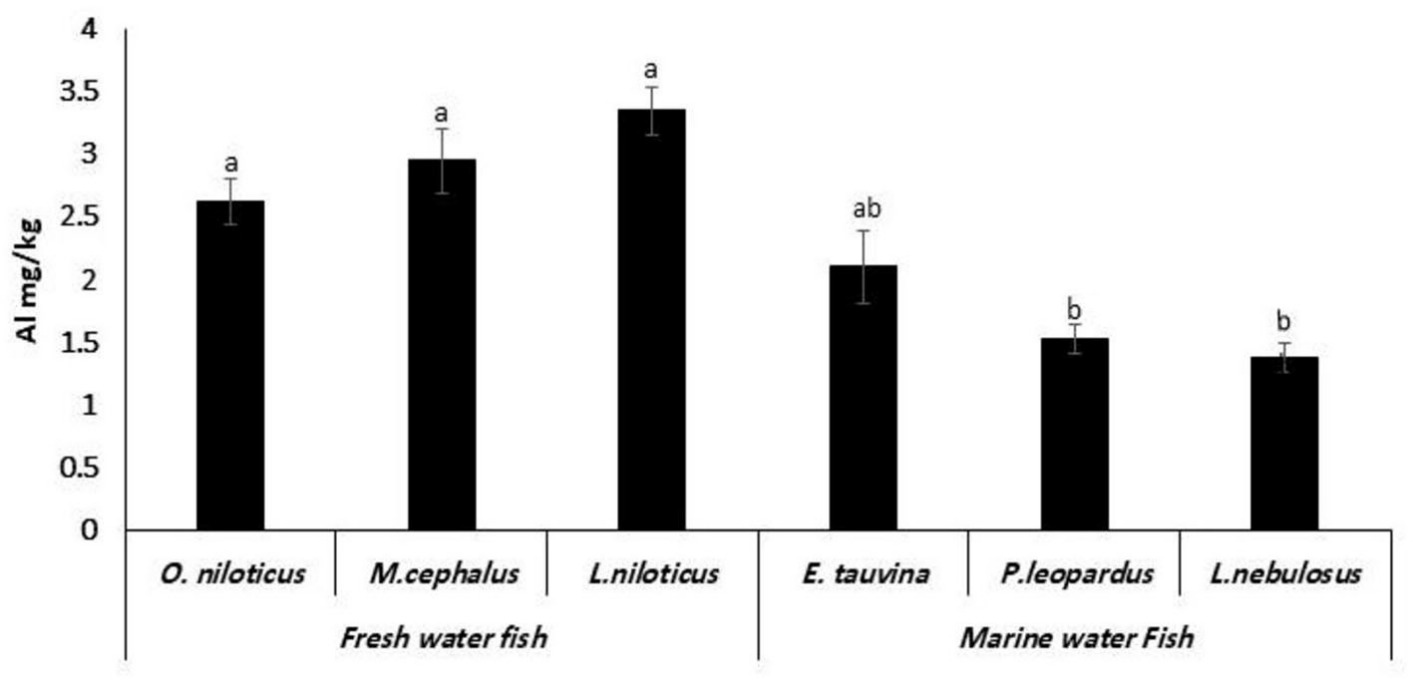
Aluminum (Al) residues (mg/kg wet weight) in fresh water and marine water fish. Data represent means ± SD (*n* = 20 for each species). ^a, b, c^Columns bearing different small letters are statistically significant at a *P*-value of < 0.05.

The Al residues were obtained in 100% of examined species. The mean values (mg/kg) were 2.62 ± 0.18, 2.95 ± 0.26, 3.35 ± 0.19, 2.11 ± 0.29, 1.53 ± 0.11, and 1.38 ± 0.12 mg/kg in examined *O. niloticus, M. cephalus, L. niloticus, E. tauvina, P. leopardus*, and *L. nebulosus*, respectively. *L. niloticus* had significantly (*P* < 0.05) the higher Al values among the examined fish samples (Figure 6).

### Public health risk assessment

Upon evaluating the acceptability of the investigated samples in comparison with the stated maximum permissible limits for the residual quantities of the heavy metals (MPL), it was clear that 2 (10%), 3 (15%), 5 (25%), 4 (20%), 3 (15%), and 5 (25%) fish samples of the examined *O. niloticus, M. cephalus, L. niloticus, E. tauvina, P. leopardus*, and *L. nebulosus* species, respectively, exceeded the recommended MPL (0.3 mg/kg wet weight) of Pb in fish according to European Commission (23), whereas 1 (5%), 8 (40%), 13 (65%), 15 (75%), 13 (65%), and 4 (20%) fish samples of the *O. niloticus, M. cephalus, L. niloticus, E. tauvina, P. leopardus*, and *L. nebulosus* species, respectively, exceeded the recommended MPL (0.05 mg/kg wet weight) for Cd. Meanwhile, only 6 (30%) of the *L. niloticus* samples exceeded the MPL 0.5 mg/kg wet weight recommended by European Commission (23) (Table 1).

**Table 1:** Acceptability of the examined fish samples according to their content of heavy metal in comparison with the European Commission Regulation.

		**Fresh water fish**			**Marine water fish**		
		***O. niloticus***	***M. cephalus***	***L. niloticus***	***E. tauvina***	***P. leopardus***	***L. nebulosus***
Pb (0.30)^a^	Within	18 (90%)	17 (85%)	15 (75%)	16 (80%)	17 (85%)	15 (75%)
	Exceed	2 (10%)	3 (15%)	5 (25%)	4 (20%)	3 (15%)	5 (25%)
Cd (0.05)^a^	Within	16 (80%)	7 (35%)	5 (25%)	7 (35%)	12 (60%)	19 (95%)
	Exceed	4 (20%)	13 (65%)	15 (75%)	13 (65%)	8 (40%)	1 (5%)
Hg (0.50)^a^	Within	20 (100%)	20 (100%)	14 (70%)	20 (100%)	20 (100%)	20 (100%)
	Exceed	-	-	6 (30%)	-	-	-

^a^European Commission (23) No 1881/2006.

The results in Table 2 showed that the EDI of various metals from fish consumption was less than the TDI μg/kg body weight for all metals.

**Table 2:** Estimated daily intake (EDI) μg/kg body weight/day of different metals in comparison with the tolerable daily intakes (TDIs) μg/kg body weight.

	**EDI (Pb)**	**EDI (Cd)**	**EDI (As)**	**EDI (Hg)**	**EDI (Al)**
*O. niloticus*	0.062	0.028	0.090	0.049	1.817
*M. cephalus*	0.076	0.056	0.187	0.187	2.046
*L. niloticus*	0.215	0.090	0.305	0.291	2.250
*E. tauvina*	0.118	0.076	0.784	0.083	1.460
*P. leopardus*	0.042	0.042	0.618	0.132	1.060
*L. nebulosus*	0.056	0.021	0.368	0.090	0.950
Average EDI	0.095	0.052	0.392	0.139	1.600
TDIs	3.570	1.000	2.100	0.228	142.860
EDI/TDI%	2.66%	5.20%	18.66%	60.96%	1.11%

Based on the anticipated daily intakes, the average THQ for all metals tested was < 1, with the exception of total As. Furthermore, with the exception of *O. niloticus*, the calculated hazard index on a total As basis was >1. Meanwhile, all evaluated species, show no risk to public health (hazard index < 1), when 10% total arsenic (inorganic arsenic only) is used as the basis for calculation. The average RI was 0.000806 due to lead exposure via fish ingestion (Table 3).

**Table 3:** Target hazard quotient (THQ) and hazard index (HI) of different metals from fish consumption and risk index (RI) of lead.

	**THQ (Pb)**	**THQ (Cd)**	**THQ(As) total**	**THQ(As) inorganic**	**THQ (Hg)**	**THQ (Al)**	**HI (As) total**	**HI (As) inorganic**	**RI (Pb)**
*O. niloticus*	0.016	0.028	0.301	0.030	0.097	0.0018	0.444	0.173	0.0005
*M. cephalus*	0.019	0.056	0.624	0.056	0.375	0.002	1.076	0.508	0.0006
*L. niloticus*	0.054	0.090	1.018	0.101	0.583	0.002	1.747	0.830	0.0018
*E. tauvina*	0.029	0.076	2.614	0.261	0.167	0.001	2.887	0.534	0.0010
*P. leopardus*	0.010	0.042	2.058	0.205	0.264	0.001	2.375	0.522	0.0004
*L. nebulosus*	0.014	0.021	1.226	0.122	0.180	0.001	1.442	0.338	0.0005
Average	0.024	0.052	1.307	0.13	0.278	0.002	1.662	0.486	0.0008

HI (As) total: Hazard index calculated depending on the obtained values of total arsenic. HI (As) inorganic: Hazard index calculated depending on the 10% of total arsenic, only inorganic, in fish (24).

## Discussion

Lead, Cd, As, Hg, and Al are examples of metals that have no known physiological functions. Owing to the nature of these metals' bioaccumulation and biomagnification, repeated ingestion of them, even at low concentrations, may have a number of toxicological effects and cause organ damage (25).

### Lead residues

Lead residues detected in fish worldwide were 0.11–1.15 mg/kg among Mediterranean sea fish species (26), 0.33–0.93 mg/kg in fish samples collected from Türkiye (27), 0.01–0.15 mg/kg in fish samples from Portugal (28), 0.11–0.89 mg/kg in fish samples from Türkiye (29), and 0.10–0.12 mg/kg in fish collected from Italian Coasts (30). The consumption of fish contributes to 2.66% of TDIs of Pb for adults (Table 2).

### Cadmium residues

The cadmium values were comparable to those of 0.006–0.024 mg/kg that were previously obtained in fish from Lake Manzala, Egypt (31), 0.003 to 0.021 μg/g i that were previously obtained n fish from Taihu Lake of China (32), and 0.07–0.10 mg/kg that were previously obtained in fish collected from Italian Coasts (30). The consumption of fish contributes to 5.20% of TDIs of Cd for adults (Table 2).

### Arsenic residues

The As residual concentration in marine fish was similar to EFSA (33) data, which reported a mean value of 1.45 mg/kg after examination of 3,505 different species. In addition, 0.78 ± 0.26 and 0.94 ± 0.31 mg/kg of salmon and trout were collected from the Norwegian fish market (34). However, As content in the current study was much higher than 0.59–0.69 mg/kg in fish collected from Italian Coasts (30). The obtained results for As freshwater fish in these study comparable to 0.22 ± 0.15 mg/kg obtained in Mexico freshwater (*Ictalurus punctatus*) (35). The As values in marine fish were significantly higher (*P* < 0.05) compared with the freshwater fish, which is attributed to the arsenic concentration being higher in seawater than in the freshwater environment (36). Another aspect that must be considered is the fact that the analytical method used in the current study only investigated total arsenic levels, although the concentration of inorganic arsenic, the most dangerous form, is of greater significance from the standpoint of food safety. However, fish and other seafood typically contain negligible amounts of inorganic arsenic (24). Arsenobetaine and arsenocholine, which are quickly and unmetabolized excreted in human urine and are regarded as being of no toxicological concern, are the main chemical forms of arsenic in seafood (37). The consumption of fish contributes to 18.66% of the TDIs of As for adults (Table 2).

### Mercury residues

The mercury residues in the current study were nearly similar to those (0.11 ± 0.22–0.19 ± 0.09 mg/kg) in fish collected from Italian Coasts (30). Meanwhile, lower Hg values were obtained at 0.014 ± 0.007 mg/kg in the Italian study (38) and 0.017 ± 0.004 and 0.020 ± 0.005 mg/kg in salmon and trout, respectively, in the Norwegian study (34). Because MeHg is harmful to the development of the brain, pregnant women and young children should be especially cautious about exposure to mercury (39). Additionally, a cautious methodology was used in this study, presuming that all mercury was present in the seafood samples as MeHg, which is significantly more poisonous than inorganic Hg, the form of mercury that is most commonly found in seafood. This strategy is consistent with research done by the EFSA (40). A panel on Contaminants in the Food Chain (CONTAM) found that MeHg typically accounts for 80–100% of the total mercury in fish muscle.

### Aluminum residues

One of the most prevalent metals in the environment and consequently in food is aluminum. However, anthropogenic activities and the acidification of the soil have caused Al levels to rise over time. Al is a well-known neurotoxic substance because it tends to build up in the brain. Numerous studies have shown a link between Al levels and various diseases, including Alzheimer's disease. Additionally, some important metals may be hampered by aluminum (41). The obtained Al values in these studies were consistent with the findings in Spain from 0.92 ± 0.71 to 3.48 ± 3.96 mg/kg (42) and in Türkiye from 0.831 to 2.228 mg/kg (43). Meanwhile, lower Al values were obtained in fish samples in the United States at 0.40 mg/kg (44). The freshwater fish samples contained significantly higher Al than marine water fish (*P* < 0.05); this may have been attributed to the higher acidity in marine water fish then the freshwater fish, which enhances the solubility of Al (45). The results demonstrate the inter-species differences in Pb, Cd, and Hg accumulation. For example, *L. niloticus* had the highest residual contents of the various metals, whereas *O. niloticus* had the lowest concentrations. The situation of the fish in the food chain may have an impact on the residual contents, as predatory fish such as *L. niloticus* fish build up higher levels of heavy metals (46). Furthermore, metal contamination of fish muscle varies by geographic region (47).

### Public health risk assessment

Regulation (EC) No. 1881/2006 (23) establishes the maximum levels of contaminants in fish as 0.5, 0.3, and 0.05 mg/kg wet weight for mercury, lead, and cadmium, respectively (Table 1). Meanwhile, there were no available regulations for As and Al worldwide adopted for fish. As a result, this study was augmented to calculate the EDI, HQ, and HI to determine the potential health risks associated with the consumption of such fish species. The obtained values of EDI (μg/kg/day) for Pb, Cd, As, Hg, and Al were 0.095, 0.052, 0.392, 0.139, and 1.60 μg/kg/day, respectively, due to fish consumption (Table 2). In addition, fish contributes 2.66, 5.2, 18.66, 60.96, and 1.11% of TDIs from Pb, Cd, As, Hg, and AL, respectively, for adults (Table 2). The readings were within the tentatively acceptable daily intakes by World Health Organization (48). The obtained THQ for Pb, Cd, Hg, and Al is below 1, suggesting that fish consumption would not carriage any health hazards due to these toxic metals. Nearly similar harmless THQ were obtained due to the consumption of fish from Italian coasts, Black Sea, and traditional fish farms in Bangladesh (30, 49, 50). However, THQ values for As greater than one are not significant for human health because the toxicological profile only refers to inorganic chemical forms of As because organic forms of arsenic are comparatively non-toxic to human health (51). According to the worst-case scenario established, 10% of the As were assumed to be inorganic (52). In the Risk Assessment Information System, the slope factor value has been given for Pb and its compounds only. If RI is >10^−4^ for all examined fish species, it indicates that lead from fish flesh constitutes a hazard to human health.

## Conclusion

The toxic metals could bioaccumulate in fish species; these elements are very harmful because they are not biodegradable and thus concentrate large amounts of them in their tissues. Therefore, the estimation of the possible health risks associated with the consumption of freshwater and marine fish is of great importance. The high values for Al and As are recorded in freshwater and marine water fish, respectively. The level of toxic metals in some fish samples exceeds the permissible limits set for these toxic metals by the European Commission Regulation. The estimated daily intakes (EDIs) of the metals were estimated as the means of Pb, Cd, As, Hg, and Al in all fish samples and the mean consumption of fish per day for adults, and the results were lower than the tolerable daily intakes (TDIs). The target hazard quotient (THQ) and hazard index (HI) of the considered metals were below the value of 1. Therefore, toxic metals in the examined fish samples do not pose health hazards to the Egyptian population based on the inorganic form of arsenic, accounting for 10% of total As. Furthermore, future studies are required for the fractionation of metallic and organic arsenic in fish and other sea foods.

## Data availability statement

The original contributions presented in the study are included in the article/supplementary material, further inquiries can be directed to the corresponding authors.

## Ethics statement

Zagazig University Institutional Animal Care and Use Committee (ZU-IACUC) for approval to use non-living animal tissues/samples in research and teaching (ZU-ICUAC/2/F/55/2023).

## Author contributions

Together, MH and NM created the study's hypothesis and conceptual framework and they also helped with the preparation of the chemicals and materials as well as the methodologies used. AM, WD, ME, FZ, ZF, and IR were involved in the experimental techniques and analysis for this study and scientific paper. The manuscript was edited and revised by all writers. All authors contributed to the article and approved the submitted version.
